# The effect of continuous care model on sleep quality in postmenopausal women: a randomized clinical trial study

**DOI:** 10.5935/1984-0063.20220059

**Published:** 2022

**Authors:** Farzaneh Akbari, Sousan Heydarpour, Nader Salari

**Affiliations:** 1 Student Research Committee, Kermanshah University of Medical Sciences, Kermanshah - Iran; 2 Department of Midwifery, Kermanshah University of Medical Sciences, Kermanshah - Iran; 3 Sleep Disorders Research Center, Kermanshah University of Medical Sciences, Kermanshah - Iran

**Keywords:** Sleep, Women, Menopause

## Abstract

**Introduction and Objective:**

Sleep disorder leads to several mental, physical, and behavioral complications. Through continuous care model, it is possible to achieve an efficient recognition of the problems of these patients and allow them to participate in solving their health issues. The effects of continuous care model on sleep quality in postmenopausal women were examined.

**Material and Methods:**

Totally, 110 postmenopausal women visiting health center in Kermanshah, Iran took part in this clinical trial. The participants were selected between May 2017 and September 2017. The participants were allocated to control and experimental groups randomly each with 55 members. The normal cares were provided to the control group, while the experimental groups took part in group consultation sessions (once every week, four session each 60-90min). Quality of sleep was examined based on Pittsburg sleep quality index before the intervention, immediately after the intervention, and a month flowing the completion of the intervention. For data analyzing, Friedman’s test, Mann-Whitney test, and chi-square test were used in SPSS.

**Results:**

A significant difference was found in the mean scores of the quality of sleep in the experimental group in three measurements occasions in the study (*p*=0.001). Despite lack of any significant difference before the intervention between the two groups, there was a significant decrease in the sleep quality score in the experimental group one month after the completion of the intervention compared to the control group (*p*<0.05).

**Conclusion:**

The continuous care model improved the sleep quality in the postmenopausal women.

## INTRODUCTION

One of the most critical stages in every woman’s life is menopause^1^. By definition, it is the final menstrual period with 12 months of amenorrhea afterward and post menopause is the time period after the final menses^2^. It is expected that the number of postmenopausal women reaches 1.2 billion by 2030^3^. In Iran, the number of women in 45-64 age range was 8.5 million in 2011 (11.33% of the total population; Iran national Statistics Organization)^4^.

The key issue about postmenopausal women and their health are vaginal urine atrophy (urogenital), vasomotor symptoms, cardiovascular diseases, osteoporosis, lower cognitive function, cancer, and sexual problems. Sleep disorders among these diseases is a serious challenge^5^,^6^ so that, poor sleep quality is very common after menopause^7^.

Sleep disorders vary in terms of prevalence so that its prevalence in premenopausal women is 16-42%, 39-47% in perimenopausal women, and 35-60% in postmenopausal women^8^. Several studies in Iran have indicated 70% prevalence of sleep disorders in women in 50-60 years age range^9^,^10^. Among the sleep disorders that are usually reported, insufficient sleep syndrome, circadian rhythm, insomnia, and obstructive sleep apnea are notable^11^. Among the symptoms that causes complaints in menopausal women are sleep-onset insomnia, early morning awakening, and frequent awakening^12^.

Post-menopausal women are prone to several sleep disorders such as obstructive sleep apnea and insomnia^13^,^14^. The latter causes concerns in these women and the prevalence of the former grows notably following menopause^15^. The prevalence of obstructive sleep apnea is in 47% to 67% range according to studies^16^,^17^.

Several factors can be named in sleep disturbances among postmenopausal women such as normal physiological changes caused by aging, postmenopausal symptoms, low health perception, stress, nervousness, mood symptoms (e.g., anxiety or depression), and comorbid chronic health issues^14^,^18^-^20^. Along with these chronobiological and biological factors, psychosocial, socioeconomic, cultural, race, and ethnicity factors can have a role in the relationship between menopause and sleep^21^,^22^.

To improve the quality of sleep, non-pharmacological and pharmacological treatment can be used. Among them is training women based on the available theories and models^23^, such as the “continuous care model” introduced by Ahmadi et al. (2001)^24^. The model has four interconnected stages namely orientation, sensitization, control, and evaluation. According to this model, the client acts as a factor of continuous care that plays a key role in their health process^23^,^25^. To implement this method, health providers need to identify patients’ problem accurately and motivate and involve them and the family in the process of solving their problems^26^. The model is aimed at designing and developing a program that leads to acceptance, higher appropriate visions, and controlling the diseases and the probable side effects^27^. This model enables health providers to determine the problems of the patients and allows them to participate in dealing with the disease^26^. An experimental study examined the impact of continuous care model on the quality of sleep in hemodialysis patients and showed that the model improved quality of sleep^28^. Another study showed that continuous care model was an effective solution to improve sleep quality in patients suffering diabetes type 2 so that the patients had a higher quality of sleep^23^.

Through this model, we can achieve a better knowledge of patients’ problem and motivate and involve them in dealing with the problem^26^. Therefore, the effects of interventions using continuous care model on sleep quality of postmenopausal women in Kermanshah health centers were examined.

## MATERIAL AND METHODS

### Study design

This was a two-group randomized clinical trial study.

### Study population

**Figure 1:**
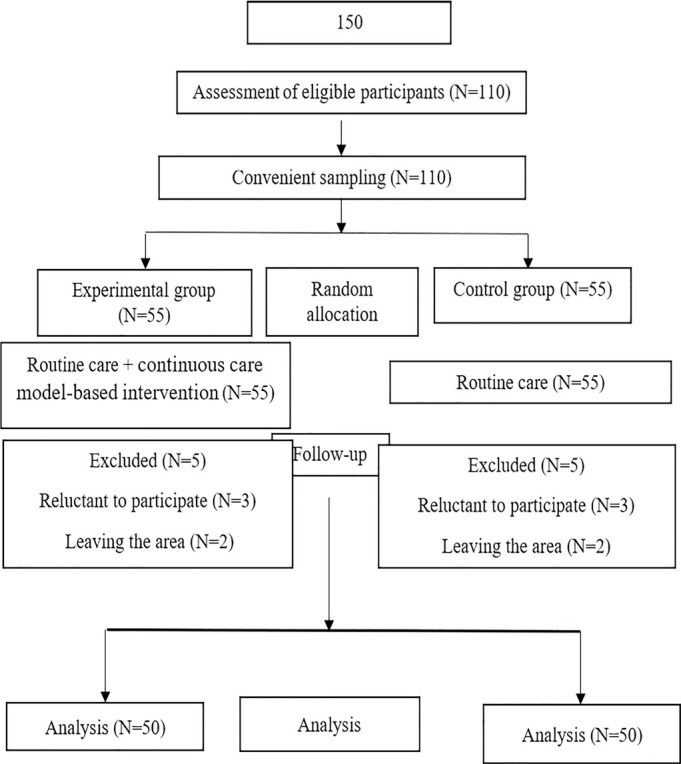
The chart of the study protocol.

The participants consisted of 110 postmenopausal women in Kermanshah health centers. The women were allocated randomly to control and experimental groups each with 55 participants (Figure 1). A simple randomization method was utilized for random allocation. Through this, 110 identical cards were used so that 55 cards were marked as “1” and the rest as “2” representing the experimental and control groups respectively. Afterward, the participants were asked to select one card and determine their group without being aware of the allocation system. The participants were selected between May 2017 to September 2017.

### Eligibility

Inclusion criteria: at least one year and at most four years past since the last menstrual cycle; no mental disease (as indicated in the medical files), no smokers, no alcohol or drug abusive use, no history of hormone therapy in the past six months, writing/reading literacy, and not using any sleep treatment (benzodiazepines, antihypertensive and so on).

Exclusion criteria: mental and physical disease, chronic pain, Alzheimer’s disease as diagnosed by the attending physician, not interested in participation, and missing more than one session.

### Intervention

Along with the standard cares provided to postmenopausal women in the health centers, the experimental group (n=55) received continuous care model-based intervention. Continuous care is designed to create mutual, effective, and continuous relationships between care-seeker and care-giver through determining patients’ problems, and sensitization for accepting permanent healthy behaviors and contributing in keeping a better health condition^24^,^25^.

The experimental group received weekly group consultations (once a week for four weeks; 60-90min) that were in the form of lecture, group discussion, answering and asking questions, and a training pamphlet. The content of sessions included the process of menopause; non-pharmacological intervention to have a better quality of sleep; why health is important, leaving behind unhealthy habits, ways to develop healthy habits such as sleeping, having longer useful sleeping time, what affects sleep, the principles of sleep hygiene and different types of sleep disorders (Table 1).

**Table 1:** Content of the session and steps of the intervention.

Model Phases	Content	Methods
Orientation (session one)	- Completing informed consent form, demographic information and Pittsburgh sleep quality index questionnaire; - Learning about the participants’ problem and creating the needed sensitivity; - Orientation with the model stages and continuing communication.	Group discussion, lecture
Sensitization (sessions two, three and four)	Discussing the process of menopause, the problems like hot flashes, overnight sweating, and non-pharmacological intervention to improve quality of sleep in the participants: - Training healthy habits and the factors that affect sleep (e.g., nutrition, exercising, physical activity, using medicines, smoking, etc.); - Training the principle of sleep hygiene and different types of sleep disorders; - Reemphasizing the importance of continuing communication; - Introducing the participants to other specialists if needed.	Consultation, group discussion, lecture, questions and answers, a training pamphlet
Control	Keeping continuous care consultation through weekly checks (telephone or visiting) depending on the care needs.	-
Evaluation	Following up, evaluating all stages and assessing the effectiveness of interventions, identifying new problems/needs of participants and continuing the care process.	-

Only the standard interventions were provided to the control group. The two groups filled out the questionnaires before the intervention, immediately after the intervention, and a month after the intervention.

To remove observer bias, the allocation of participants, and whole process of administering the questionnaires were carried out by an outsider with no role in the study.

### Sampling techniques

Based on Mehdizadeh et al. (2010)^29^ and with δ1=5.12, µ1=15.31, δ2=5.3, µ2=11.86, α=0.05, and β=0.1, the estimated required sample size was 48. Assuming probable attrition, each group was comprised of 55 participants. To select the clinics, four regions were defined in Kermanshah City based on socioeconomic status with 20 clinics. Afterward, a clinic was selected randomly from each area and the participants were selected based on quotas for each clinic. Eventually, the participants were allocated randomly to both groups (each with 55 members).

### Study instrument

For collecting data, a demographics checklist and the Pittsburgh sleep quality index (PSQI) were used. The PSQI was introduced by Buysse et al. (1989)^30^ to measure attitude of the respondent about sleep quality in the past one month. The tool includes nine items and item No. 5 contains 10 secondary items so that there are totally 19 items in the tool. The items are based on Likert’s four-point scale (0-3) with seven subscales namely sleep latency, habitual sleep efficiency, subjective sleep quality, sleep duration, use of sleeping medication, sleep disturbances, and daytime dysfunction. The answer to each item is scored from 0 to 3 and the score range is from 0 to 21. There was a negative relationship between the score and sleep quality. Score higher than 6 indicates undesirable quality of sleep^31^.

The Persian version of the instrument was examined in terms of validity and reliability by Chehri et al. (2020)^32^, with Cronbach’s Alpha and correlation coefficient equal to 0.81 and 0.89, respectively.

### Statistical analysis

For data analyzing, descriptive statistics (viz. mean, frequency, and standard deviation) and analytical tests (viz. Mann-Whitney and chi-square) were used in SPSS^24^. Kolmogorov-Smirnov (KS) test was utilized to ascertain normality of distribution of the data. Friedman’s test (non-parametric equivalent of frequent measure test) was utilized to survey mean score trends of the quality of sleep and its sub-scales before the intervention, immediately after the intervention, and a month following the intervention in the experimental group.

Mann-Whitney tests was used for comparing the mean scores of sleep quality (total score) and the subscales before, immediately after, and one month after in the experiment and control groups. The normality of the data was examined using KS test. The significance level for all tests was less than 0.05.

### Ethics

As to ethical concerns, the objectives of the study were explained to the participants and they expressed their consent to participate in a written form. The place and time of the sessions were determined to the convenience of the participants and confidentiality of the information was observed. The participants volunteered and were allowed to leave the study at will. After the study, the participants in the control group received the same educational contents similar to the experimental group. All services beyond the knowledge of the authors were provided by physicians.

The study was approved by the ethics committee under the No. KUMS.RES.1395.754 and registered on Iran Clinical Trial Website (IRCT2017042614333N73).

## RESULTS

Totally, 110 postmenopausal women took part in the study and among them ten participants left the study (five from the experimental group and five from the control group who were reluctant to participate or moved from the area). Eventually, 100 postmenopausal women including 50 in the intervention group and 50 in the control group completed the study.

There were no significant differences between the groups in terms of demographical variables (*p*>0.05) (Table 2).

**Table 2:** Individual characteristics of the menopausal women in the experimental and control group.

Variable		Experimental group N=55	Control group N=55	*p-*value
Women’s age		53.62±2.88	53.55±3.29	0.618^*^
Time interval from menopause		2.22±1.07	2.29±1.27	0.782^*^
Mother’s education level	Under diploma	43(86)	38 (76)	0.202^**^
	Diploma and academic	7(14)	12(24)	
Mother’s marriage status	Married	45(90)	45(90)	0.999^**^
	Single parent	5(10)	5(10)	
Income level	Less than three million IRR	2(4)	7(14)	0.08^***^
	ten to three million IRR	48(96)	43(86)	
Residence	Urban	48(96)	50(100)	0.247^***^
	Rural	2(4)	0(0)	
Children’s number	1-2	5(10)	9(18)	0.502^****^
	3-4	36(72)	32(64)	
	≥5	9(18)	9(18)	
Having an addict member in the family	Yes	1(2)	1(2)	0.753^***^
	No	49(98)	49(98)	
BMI	<18.5	8(16)	6(12)	0.696^****^
	18.5-24.9	22(44)	26(52)	
	>25	20(40)	18(36)	

Captions:

* Mann-Whitney test;

** Yates Correction Test;

*** Fishers exact test;

**** Chi-square test.

The KS test indicated that the variable age was not distributed normally. The mean age of the women in the experimental and control groups were 53.55±3.29 and 53.62±2.88, respectively; so that the two groups had the same age distribution (*p*=0.618).

In addition, time interval of menopause in the experimental group and control group were 2.22±1.07 and 2.29±1.27, respectively; which means no significant difference existed between the two groups (*p*=0.618).

Homogeneity of variables education level and marriage status was supported by Yates correction test (*p*>0.05). Fisher’s exact test supported the homogeneity of demographical specifications (viz. income level, residence, and having an addict member in the family) (*p*>0.05). To test homogeneity of the number of children and body mass index (BMI), chi-squared test was used, which supported the homogeneity of the variable in the two groups (*p*>0.05).

The results of Wilcoxon post hoc test for double comparison between different time periods in the experimental group indicated a significant difference in terms of the mean score of overall sleep quality before and immediately after the intervention (*p*=0.001). Moreover, there was a significant difference between the mean score of overall sleep quality before and one month after the intervention (*p*=0.001).

Friedman’s test showed that the mean score of quality of sleep and the subscales (except for the use of sleeping medications subscale) in three intervals of the study (before intervention, immediately after intervention, and one month after the intervention) were significantly different in the experimental group (*p*=0.001). In addition, the results of Friedman’s test showed that, in the control group, the mean scores of overall sleep quality and its subscales including habitual sleep efficiency, use of sleeping medications, sleep duration, sleep disturbances, and daytime dysfunction in three intervals of the study (before the intervention, immediately after the intervention, and one month after the intervention) were not different significantly (*p*=0.080). Still, the mean scores of subjective sleep quality significantly degraded a month after the intervention (*p*=0.001) and the sleep latency means scores showed significant improvement before the intervention and immediately after the intervention (*p*=0.041).

Mann-Whitney test showed no significant difference between the mean score of sleep quality and the subscales (except for the sleep disturbances subscale) in the two groups before the intervention (*p*>0.05). However, the experimental group demonstrated a significantly lower scores in the quality of sleep and its subscales (except for the sleep duration, sleeping medications, sleep disturbances subscales) one month following the intervention compared to the control group (*p*<0.05) (Table 3).

**Table 3:** Comparison of mean and standard deviation of total score and subscales of sleep quality between experimental and control groups.

Sleep quality/Time	Experimental group	Control group	p-value
**Subjective sleep quality**			
Before	0.64±1.78	0.55±1.88	0.341^**^
Immediately after	0.57±1.44	0.59±1.88	0.001^**^
One month after	0.54±0.54	0.68±2.16	0.001^**^
*p*-value	0.001^*^	0.001^*^	
**Sleep latency**			
Before	1.78±0.81	1.78±0.88	0.927^**^
Immediately after	1.56±0.78	1.60±0.90	0.900^**^
One month after	0.9±0.67	1.68±0.86	0.001^**^
*p*-value	0.001^*^	0.041^*^	
**Sleep duration**			
Before	0.62±0.83	0.36±0.48	0.213^**^
Immediately after	0.38±0.60	0.44±0.70	0.727^**^
One month after	0.22±0.41	0.36±0.48	0.125^**^
*p*-value	0.001^*^	0.779^*^	
**Habitual sleep efficiency**			
Before	0.72±1.03	0.40±0.67	0.166^**^
Immediately after	0.26±.56	0.42±0.78	0.391^**^
One month after	0.080±0.27	0.32±0.58	0.015^**^
*p*-value	0.001^*^	0.529^*^	
**Sleep disturbances**			
Before	1.60±0.53	1.30±0.46	0.004^**^
Immediately after	1.48±0.54	2.16±6.48	0.044^**^
One month after	1.34±0.47	1.22±0.46	0.220^**^
*p*-value	0.001^*^	0.074^*^	
**Use of sleeping medications**			
Before	0.00	0.00	0.00
Immediately after	0.00	0.00	0.00
One month after	0.00	0.00	0.00
*p*-value	0.00	0.00	
**Daytime dysfunction**			
Before	1.78±0.64	1.56±0.54	0.906^**^
Immediately after	1.48±0.57	1.5±0.58	0.713^**^
One month after	1.08±0.52	1.56±0.57	0.001^**^
*p*-value	0.001^*^	0.607^*^	
**Overall sleep quality**			
Before	8.40±3.09	7.44±1.98	0.140^**^
Immediately after	6.76±2.20	8.16±6.75	0.168^**^
One month after	4.28±1.79	7.5±2.10	0.001^**^
*p*-value	0.001^*^	0.080^*^	

Notes:

* Friedman’s test;

** Mann-Whitney tests.

## DISCUSSION

The effect of continuous care model on the sleep quality in postmenopausal women was studied. The intervention improved significantly the overall sleep quality and its subscales (except for the sleep duration, sleeping medications, and sleep disturbances subscales). Our results are in agreement with studies about continuous care model and its effect on sleep quality of victims of chemical weapons^29^, diabetics^27^, and hemodialysis patients^26^.

Applying continuous care model can effectively decrease the mean scores of sleep quality one month after the intervention; still, there was not such trend in the control group. The findings are in agreement with the results of other studies^26^,^27^,^29^. To elaborate on the findings and the effects on the sleep quality in postmenopausal women, patients in the model are considered as the focus of a continuing care. The care is a normal and mutual process to establish effective interactions between the caregiver and care provider. The objective is to realize the necessity and problems of patients, sensitize them about cultivating continuous healthy behaviors, and enable them to keep their recovery and improve their health^33^. The model is based on empowering the patients and changing life style^27^. In addition, in the current study, to have a higher quality of sleep in the participants, the interventions were all non-pharmacological and the needed educations as to observing sleep quality were provided to the participants. Among them, quitting bad habits, choosing good pre-bedtime habits, increasing useful sleep time, the factors in sleep (exercising, nutrition, smoking, and medicines), and the standards of sleep hygiene (asleep and awake time, nutrition, stimulators, use of drugs, physical activity, and sleep environment) are notable.

As the findings showed, the intervention through continuous care model improved the subscales of quality of sleep such as sleep latency, habitual sleep efficiency, daytime dysfunction, and subjective sleep quality. Another study showed that there was a significant difference after intervention using continuous care model between control and experimental groups as to subjective sleep quality, daytime dysfunction, and total score of quality of sleep^29^. However, the intervention using continuous care model did not improve the subscales of sleep duration, use of sleeping medications, and sleep disturbances. A study was carried out on the effects of continuous care model on quality of sleep type 2 diabetic individuals. The researchers concluded that the continuous care model did not improve the quality of sleep and its subscales and the changes in the control and experimental groups were insignificant. To elaborate on the inconsistent findings, different study population and special diseases (chemical weapon victims) with sleep disorder are notable. In addition, inclusion criteria were different between the two studies. For instance, as to the inclusion criteria, there were no sleep medicine (such as antihistamines and antihypertensive). This shows the reason for failure of the model in improving the subscale using sleep medicine. In addition, in the present study, the experimental group had significantly higher scores for sleep disturbances before intervention, compared to the control group.

Furthermore, the mean scores of subjective sleep quality significantly degraded one month after the intervention and the sleep latency means scores showed a significant improvement between the intervals before intervention and immediately after the intervention in the control group. It is evident that the gradual changes and the standard cares in the health centers were effective in the control group; which means having more experience led to a better sleep latency in the postmenopausal women of the control group.

The mean scores of overall sleep quality did not change immediately following the intervention. This is consistent with another study about the effectiveness of continuous care model^26^. However, incongruent with the results of other studies^28^,^34^. Clearly, nature of the model and the specifications of the participants explain the findings. It appears that the model is a dynamic model and the instructions of the models can help the users to use the benefits of the model. Therefore, the effect of the model on the user appears gradually with patience and continuous efforts of care givers^26^,^28^,^34^. That is, the demographical specifications of our participants and those of other studies were not the same and different results were not unexpected.

Among the advantages of this study, examining the participants in the two groups twice after the intervention is notable. In addition, randomization and the implementation of consultation sessions were of other advantages. Moreover, a standard tool normalized for Iranian population was used.

One of the limitations of this study was the impossibility of blinding the study. In addition, the follow-up was only one month, which is another limitation of the study. Different results can be expected with a longer follow-up. Moreover, the intervention group had significantly higher scores for sleep disturbances before intervention, compared to the control group. Investigating the effectiveness of continuous care model-based intervention without this limitation is recommended.

## Conclusion

Given the obtained results of the present study, implementation of the continuous care model improved sleep quality in the postmenopausal women. Thus, the model can be used to help postmenopausal women having a better sleep quality.
